# Beneficial Effects of Dietary Flaxseed Oil through Inflammation Pathways and Gut Microbiota in Streptozotocin-Induced Diabetic Mice

**DOI:** 10.3390/foods12173229

**Published:** 2023-08-28

**Authors:** Hui Xia, Ying Wang, Xiangling Shi, Wang Liao, Shaokang Wang, Jing Sui, Guiju Sun

**Affiliations:** 1 Key Laboratory of Environmental Medicine and Engineering of Ministry of Education, Department of Nutrition and Food Hygiene, School of Public Health, Southeast University, Nanjing 210009, China; huixia@seu.edu.cn (H.X.); 220223682@seu.edu.cn (Y.W.); sxl1573@126.com (X.S.); wangliao@seu.edu.cn (W.L.); shaokangwang@seu.edu.cn (S.W.); suijing@nuist.edu.cn (J.S.); 2 Research Institute for Environment and Health, Nanjing University of Information Science and Technology, Nanjing 211544, China

**Keywords:** flaxseed oil, type 1 diabetes, hepatic inflammation, gut microbiota, TLR4/MyD88

## Abstract

Flaxseed oil (FO) has displayed potential anti-diabetes properties by providing a high content of α-linolenic acid. However, the effects and mechanisms of FO on type 1 diabetes are still unclear. The present study aims to explore the effects of different doses of FO feeding on hepatic inflammation and gut microbiota in streptozotocin-induced diabetic mice. Forty-eight six-week-old C57BL/6J male mice were divided into a control group (CON), a diabetic group (MOD), a diabetes with 7.0% *w*/*w* FO feeding group (FO-L), and a diabetes with 10.5% *w*/*w* FO feeding group (FO-H) for six weeks. The 7.0% *w*/*w* and 10.5% *w*/*w* FO feeding groups exhibited potential recovery of the number and size of pancreas tissues. The fasting blood glucose level was significantly decreased only after 4 weeks of feeding with 10.5% *w*/*w* FO in diabetic mice. The 10.5% *w*/*w* FO feeding group significantly decreased the postprandial blood glucose level of mice in the OGTT test. Hepatic glycogen levels were dramatically upregulated in the mice fed with both 7.0% *w*/*w* and 10.5% *w*/*w* FO. FO feeding significantly attenuated hepatic LPS, TNF-α, and IL-1β levels. In addition, we observed that 7.0% *w*/*w* and 10.5% *w*/*w* FO feedings notably downregulated hepatic gene and protein expressions of TLR4, MyD88, and P65. Furthermore, only 10.5% FO regulated fecal microbiota by increasing the relative abundance of the Bacteroidetes phylum, Lactococcus family, and Muribaculaceae and Streptococcaceae family and genus in streptozotocin-induced diabetic mice. Therefore, we conclude that FO feeding plays a role in anti-inflammation via the regulation of hepatic LPS/TLR4/MyD88 pathways and gut microbiota. In addition, different doses of FO supplementation may exhibit varying mechanisms in streptozotocin-induced mice.

## 1. Introduction

Type 1 diabetes is characterized by deficient insulin production mediated by immunity that could occur at any age. By 2017, 9 million people had been diagnosed with type 1 diabetes and most of them were in high-income countries [1]. The incidence of type 1 diabetes has increased by 3%–4% over the past three decades and is closely associated with environmental factors such as childhood obesity, vitamin D, n-3 polyunsaturated fatty acid, and dietary sugars [2]. Immune suppression and modulation still cannot explain all issues caused by type 1 diabetes so far, which was not expected from traditional views. Recently, plenty of evidence has agreed that inflammation plays an important role in beta-cell dysfunction [3]. Furthermore, inflammation also initiates proficient metabolic, epigenetic, and self-antigenic variations, leaving beta cells exposed to the immune system by exacerbating autoimmune dysregulation [4]. Therefore, it provides us with a new viewpoint that type 1 diabetes could be improved by reversing immune-related inflammation cytokines. In the nutrition area, many functional food ingredients or bioactive components demonstrate inflammation and immune modulation. Among the above factors, an n-3 polyunsaturated fatty acid deficiency could aggravate the progress of type 1 diabetes by heightening inflammatory reactions [5]. Flaxseed oil, as the main content of flaxseed, is composed of low saturated fatty acid, moderate monounsaturated fatty acid, and enriched polyunsaturated fatty acid [6]. Flaxseed oil as a functional food ingredient has displayed potential health benefits because of its rich source of n-3 polyunsaturated fatty acid by providing a high content of α-linolenic acid (ALA) [7]. A lower intake of ALA was observed in patients with type 1 diabetes compared with a healthy population [8], which indicated the vital role of ALA in the progress of type 1 diabetes.

Numerus epidemiological publications have reported the relationship between ALA intake and chronic diseases. Dietary ALA intake was inversely associated with plasma triacylglycerol in a cross-sectional study of 4440 white subjects, indicating a reduction in cardiovascular disease risk [9]. The dietary intake of ALA (1.25 ± 0.07 g) among adults was positively associated with lower odds of peripheral neuropathy [10]. The prospective data in the Nurses’ Health Study indicated that increasing the dietary intake of ALA may reduce the risk of sudden cardiac death [11]. A high consumption of dietary ALA was positively associated with higher plasma insulin in non-diabetic subjects [12]. A meta-analysis showed that every 1 g/d increase in ALA intake was linearly associated with a 12% decrease in fatal cardiovascular disease risk [13]. Another study that included ten subjects with type 2 diabetes found that 5 g of linseed oil exchange improved the coagulation function (including the plasmin α2–plasmin inhibitor complex, plasminogen activator inhibitor-1 activity, and thrombin antithrombin III complex) [14]. A higher intake of ALA was negatively associated with cardiac events (HR = 0.58; 95% CI, 0.39–0.85) in type 2 diabetes in a prospective cohort study [15]. Lower percentages of ALA in RBC membranes were associated with worse glucose metabolism with the follow-up in a diabetes cohort [16]. ALA consumption was associated with a decreased risk of type 2 diabetes in Chinese Singaporeans [17]. A systematic review of 83 randomized controlled trials indicated that increasing the ALA intake may increase fasting insulin by 7% [18]. An intake with supplementations of 2 g and 1 g of linoleic acid and ALA combined with restricted energy could help control pre-diabetes of young obese women, respectively [19]. However, the results were inconsistent. A prospective cohort study from the Australian Longitudinal Study on Women’s Health found that ALA intake was positively associated with the incidence of type 2 diabetes (RR = 1.84; 95% CI, 1.25–2.71) after 6 years of follow-up [20]. Milled flaxseed and flaxseed oil intake did not affect glycemic control in adults with well-controlled type 2 diabetes [21]. Overall, ALA and flaxseed oil intake decreased chronic disease incidence and mortality. However, the relationship between ALA or flaxseed oil intake and diabetes, especially for type 1 diabetes, is still unclear.

Few studies uncovering the mechanisms between ALA or flaxseed oil and type 1 diabetes have been conducted by researchers. An in vivo study reported that ALA and linoleic acid reduced pancreas damage, insulin, and glucose plasma levels, and restored Δ6 desaturase activity and mRNA expression levels in streptozotocin (STZ)-induced diabetes in mice [22]. ALA supplementation for 5 months partially regulated the physicochemical properties of mitochondrial membranes and helped control glycemia in rats with type 2 diabetes [23]. ALA and gamma-linolenic acid synergistically interacted to improve the nitric-oxide-mediated neurogenic and endothelium-dependent relaxation of the corpus cavernosum in STZ-induced rats [24]. ALA prevented diabetes-induced endothelial dysfunction by enhancing eNOS activity and attenuated oxidative/nitrative stress by inhibiting iNOS and NADPH oxidase expression and ONOO- production [25]. Overall, ALA has shown potential anti-diabetes properties.

It is clear that inflammation is related to the progression of type 1 diabetes [2]. Publications have displayed that ALA supplementation trials demonstrated its beneficial effects on anti-inflammation in individuals with obesity [26,27]. ALA feeding restricted T-cell-driven inflammation in apolipoprotein E knockout mice [28]. Diets enriched with ALA and ALA supplement reduced oxidative stress and inflammation of myocardial infarction, colitis, allergy, and colorectal cancer in rodents [29,30,31,32]. However, whether ALA supplementation could act as an anti-inflammatory and its underlying mechanisms in type 1 diabetes individuals or rodents has not been clearly clarified yet. Furthermore, scientists have recognized new pathways that may mediate diabetes by altering human microbiota. It has been found that Bacteroidetes dominated at the phylum level, butyrate-producing bacteria lacked functional diversity, and community stability reduced in individuals with preclinical Type 1 diabetes [33]. ALA feeding regulated white adipose tissue, liver, and intestinal homeostasis in mice fed a high-fat diet, which may be related to gut microbiota [34]. Flaxseed oil supplementation modulated gut dysbiosis, short-chain fatty acids, and bile acids in atherosclerosis mice [35]. However, the effects of ALA-enriched flaxseed oil on gut microbiota in type 1 diabetes is still unclear. Therefore, the present study aimed to explore whether ALA-enriched flaxseed oil feeding could regulate inflammation and gut microbiota in mice with Type 1 diabetes.

## 2. Materials and Methods

### 2.1. Animals

The animal study protocol was in accordance with the Animal Management Committee and Animal Ethical Committee of Jiangsu Province and was approved by the Animal Experimental Ethical Committee of Southeast University (NO: 20190928016). Forty-eight six-week-old C57BL/6J male mice were purchased from Shanghai SLAC Laboratory Animal Company and housed in the Specific Pathogen-Free (SPF) animal laboratory at a room temperature of 20–26 °C and relative humidity of 45–60% with a 12 h light/dark cycle.

### 2.2. Study Design

Type 1 diabetes was established by the intraperitoneal injection of a large dose of streptozocin (170.00 mg/kg, STZ). A fasting blood glucose level ≥ 16.7 mmol/L was considered as qualified Type 1 diabetic mice. Flaxseed oil was added in the diet and the details was shown in the supplementary materials. Mice were divided into a control group (CON), a diabetes group (MOD), and diabetes plus flaxseed oil groups (7.0 g/100 g flaxseed oil (FO-L) and 10.5 g/100 g flaxseed oil (FO-H) added in the diet). Twelve mice were allocated to each group. Mice in CON and MOD groups were fed with the chow diet, and mice in FO-L and FO-H groups were fed with the diet added with 7.0% *w*/*w* and 10.5% *w*/*w* flaxseed oil for six weeks. The tail veil blood glucose levels of 0 min, 30 min, and 120 min were recorded and then Oral Glucose Tolerance Test (OGTT) was conducted after 2 g/kg of body weight of glucose was fed intragastrically. Mice were sacrificed with deep isoflurane. Liver tissues, pancreas, and fecal samples were collected, snap-frozen in liquid nitrogen, and stored at −80 °C for subsequent analysis.

### 2.3. Inflammatory Cytokine Detection, Western Blotting, and RT-PCR Analysis

Tumor necrosis factor-α (TNF-α), interleukin-1β (IL-1β), lipopolysaccharide (LPS), and IL-6 in the liver tissues were examined using the enzyme-linked immunosorbent assay (Elisa, Nanjing Jiancheng Bioengineering Institute) according to the manuscript. 

Total RNA of the liver tissues was extracted using TRIGene (Cat: P118, GenStar, Beijing, China), and the reverse transcription of RNAs to cDNA was performed using StarScript III One-Step RT-PCR Kit (Cat: A236, GenStar, Shanghai, China). The 2× RealStar Fast SYBR qPCR Mix (Cat: A301, Genstar, Shanghai, China) was used for the detection of cDNA target sequences after RNA reverse transcription. A Real-Time PCR System (788BRO-7517, Bio-Rad, Hercules, CA, USA) equipped with the CFX Maestro 1.1 (version 4.1.2433.1219) software was applied to perform the analysis. The reaction was performed at 95 °C for 2 min, followed by 40 cycles of 95 °C for 15 s, 60 °C for 15 s, and 72 °C for 30 s. The dissociation curve was detected from 60 to 95 °C. The house-keeping gene β-actin was used as a control, the β-actin sense primer was 5′-CGTTGACATCCGTAAAGACC-3′, and the reverse primer was 5′-AACAGTCCGCCTAGAAGCAC-3′. The equation 2^−ΔΔCt^ was calculated to show the relative mRNA levels. 

Antibodies were purchased from Cell Signaling Technology: primary antibodies for Toll-like receptor 4 (TLR4), myeloid differentiation factor 88 (MyD88), p65 (nuclear factor κB), Toll Like Receptor Adaptor Molecule 2 (TICAM2), and β-actin. After homogenization of liver tissues, protein samples were separated using 10% PAGE Gel Fast Preparation Kit (Cat: PG112, Shanghai Epizyme Biomedical Technology Co., Ltd., Shanghai, China) at 90 V for 90 min and then transferred onto nitrocellulose membranes with Omni-Flash™Western Blot Rapid Transfer Buffer (Cat: PS201S, Shanghai Epizyme Biomedical Technology Co., Ltd., Shanghai, China) for 15 min at 400 mA. Then, membranes were blocked in Protein-Free Rapid Blocking Buffer (Cat: PS108P, Shanghai Epizyme Biomedical Technology Co., Ltd., Shanghai, China) for 30 min, and immunoblotting was conducted through incubation with the primary antibodies for more than 12 h at 4 °C. After incubation with HRP-conjugated IgG (Sangon Biotech Shanghai, Co., Ltd., Shanghai, China) for 1 h at room temperature, immunoreactive bands were visualized using a chemiluminescent substrate (Millipore, Darmstadt, Germany) and analyzed using the Gel Imaging System (Cat: 5300, Tanon Science & Technology, Co., Ltd., Shanghai, China).

### 2.4. 16S rDNA Sequencing Analysis

PowerSoil® DNA Isolation Kit (MO BIO, Carlsbad, CA, US) was used to extract the fecal DNA. We then detected PCR amplification and purification of V3 + V4 variable regions of DNA (F: 5’-ACTCCTACGGGAGGCAGCA-3’, R: 5’- GGACTACHVGGGTWTCTAAT-3’). The supernatant of amplified products was transferred to enzyme-free EP tubes, which were quantified using the Solexa PCR system. Microbial 16S rDNA was sequenced on the Illumina Hiseq 2500 platform (Norcross, GA, USA). The Flash software (version 1.2.11), Trimmomatic software (version 0.33), and UCHIME software (version 8.1) were applied to form raw data. We used Trimmomatic software (version 0.33) and UCHIME software (version 8.1) to obtain the OTU (operational taxonomic unit) number of each sample. Metastats software was used to determine differences between the groups.

### 2.5. Statistical Analysis

All data are presented as means ± SEM. One-way ANOVA was performed to analyze the above data with the significant difference defined as *p* value < 0.05. GraphPad Prism 9.2 was used to perform all analyses.

## 3. Results

### 3.1. Flaxseed Oil Altered Glucose, Lipid Metabolism, and Inflammation in Type 1 Diabetic Mice

**Figure 1:**
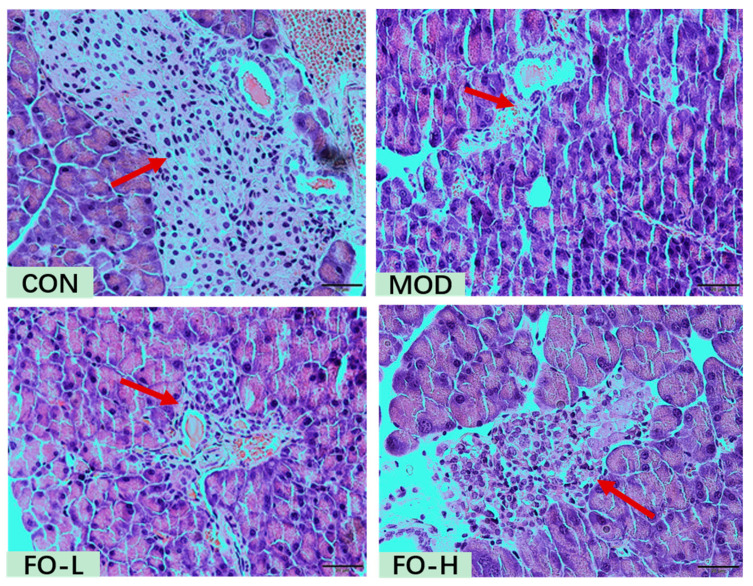
Histopathological observation of flaxseed oil fed on pancreas of STZ-induced type 1 diabetic mice. CON, the control group; MOD, the diabetic group; FO-L, 7.0% *w*/*w* flaxseed-oil-added group; FO-H, 10.5% *w*/*w* flaxseed-oil-added group.

The histology of pancreas tissues was evaluated, as shown in Figure 1. The pancreas showed potential recovery when treated with flaxseed oil (7.0% *w*/*w* and 10.5% *w*/*w*). The number and size of the pancreas tissue exhibited both in FO-L and FO-H groups increased more than those in the MOD group.

**Figure 2:**
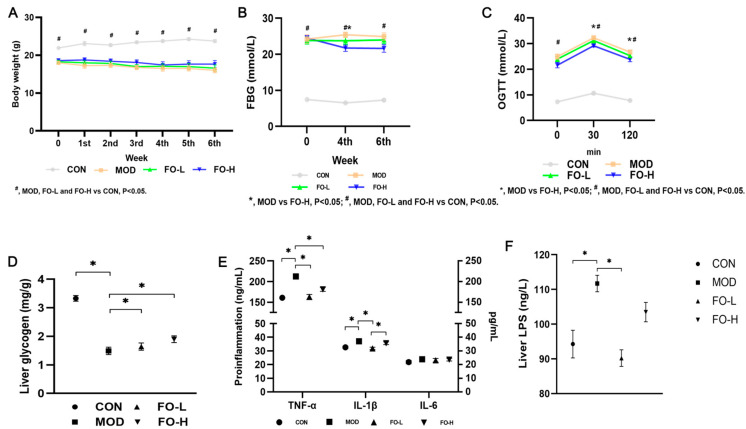
Effects of flaxseed oil feeding on hepatic and circulating biochemical parameters. (**A**) Body weight; (**B**) fasting vein tail blood glucose level; (**C**) OGTT test; (**D**) liver glycogen level; (**E**) inflammation indexes; (**F**) liver LPS levels. FBG, fasting blood glucose; OGTT, Oral Glucose Tolerance Test; TNF-α, Tumor necrosis factor-α; IL-1β, interleukin-1β; LPS, lipopolysaccharide; CON, the control group; MOD, the diabetic group; FO-L, 7.0% *w*/*w* flaxseed-oil-added group; FO-H, 10.5% *w*/*w* flaxseed-oil-added group. *, *p* < 0.05 in Figure 2D–F.

The body weight of mice did not show a significant change in flaxseed-oil-treated groups (Figure 2A). For 6 weeks of intervention, the fasting blood glucose levels (FBG) of mice in the FO-H group showed a significant decrease only after 4 weeks of intervention compared with the MOD group (Figure 2B). Furthermore, only mice treated with 10.5 g/100 g of flaxseed-oil-added diet showed significantly decreased glucose levels compared to the MOD group when conducting the OGTT test (Figure 2C). Flaxseed oil upregulated liver glycogen in both FO-L and FO-H groups with significance (Figure 2D). 

In addition, flaxseed oil feeding exhibited anti-inflammation potential by decreasing liver TNF-α and IL-1β levels (Figure 2E) in Type 1 diabetic mice. Flaxseed oil attenuated liver LPS levels in both intervention groups (Figure 2F).

### 3.2. Flaxseed Oil Changed TLR4/MyD88 Pathway in Type 1 Diabetic Mice

**Figure 3:**
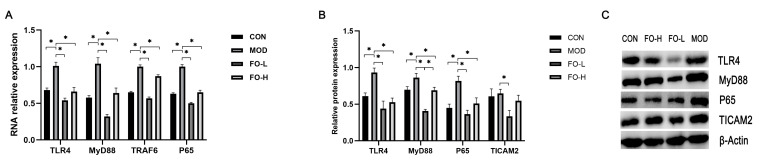
Effects of flaxseed oil feeding on hepatic-inflammation-related gene and protein expression in streptozotocin-induced mice. (**A**) Gene expression ratio; (**B**,**C**) protein expression ratio and Western blot bands. TLR4, Toll-like receptor 4; MyD88, myeloid differentiation factor 88; P65, nuclear factor κB; TRAF6, TNF-receptor-associated factor 6; TICAM2, Toll Like Receptor Adaptor Molecule 2; CON, the control group; MOD, the diabetic group; FO-L, 7.0% *w*/*w* flaxseed-oil-added group; FO-H, 10.5% *w*/*w* flaxseed-oil-added group. *, *p* < 0.05.

The TLR4/MyD88 pathway was then examined at gene and protein levels. Both 7.0% *w*/*w* and 10.5% *w*/*w* flaxseed-oil-added diets significantly downregulated the gene expression of TLR4, MyD88, TRAF6, and P65 compared to those mice fed with HFD in the MOD group (Figure 3A). Both 7.0% *w*/*w* and 10.5% *w*/*w* flaxseed-oil-added diets downregulated the protein expression of TLR4, MyD88, and P65 compared to those mice fed with HFD in the MOD group (Figure 3C). However, only the 7.0% *w*/*w* flaxseed-oil-added diet significantly decreased the protein expression of TICAM2 compared to mice fed with HFD in the MOD group.

### 3.3. Flaxseed Oil Regulated Fecal Microbiota in Type 1 Diabetic Mice

**Figure 4:**
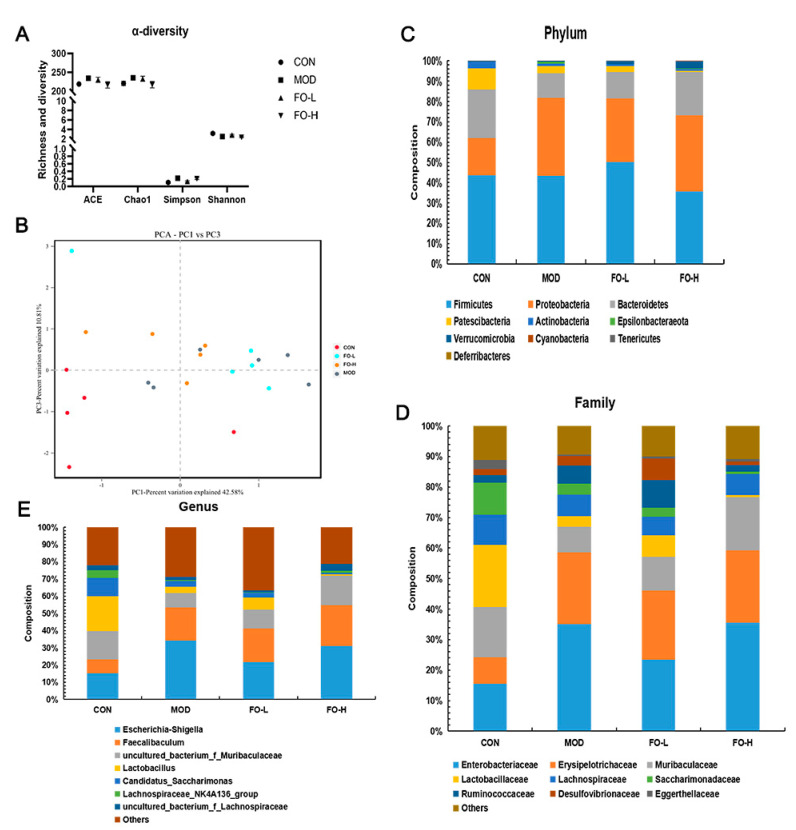
Effects of flaxseed oil feeding on the distribution of gut microbiota in streptozotocin-induced mice. (**A**) α-diversity regulation; (**B**) PCA (principal component analysis) among four groups; (**C**–**E**) microbiota distribution effects of flaxseed oil at phylum, family, and genus levels. CON, the control group; MOD, the diabetic group; FO-L, 7.0% *w*/*w* flaxseed-oil-added group; FO-H, 10.5% *w*/*w* flaxseed-oil-added group.

16S rDNA Sequencing was performed to examine and calculate the composition of fecal microbiota. Flaxseed oil did not significantly change the α-diversity (ACE, Chao1, Simpson, and Shannon indexes) of fecal microbiota (Figure 4A). β-diversity was evaluated using PCA analysis and the results showed that FO-L, FO-H, and MOD groups were slightly separate, which indicated that flaxseed oil feeding may change the overall phyla distribution in feces with the 42.58% contribution of PC1 and 10.81% contribution of PC3 (Figure 4B). We then explored the composition of fecal microbiota at phylum, family, and genus levels. Firmicutes, Proteobacteria, Bacteroidetes, and Patescibacteria were the dominant bacteria at the phylum level and comprised more than 95% in all groups (Figure 4C). At the family level, nine bacteria were dominated by nine kinds of phyla bacteria, including Enterobacteriaceae, Erysipelotrichaceae, Muribaculaceae, Lactobacillaceae, Lachnospiraceae, Saccharimonadaceae, Ruminococcaceae, Desulfovibrionaceae, and Eggerthellaceae (Figure 4D). In addition, seven kinds of phyla were dominant at the genus level: *Escheichia-Shigella, Faecailbaculum*, *uncultured_bacterium_f_Muribaculaceae*, *Lactobacillus*, *Candidatus_Saccharimonas*, *Lachnospiraceae_NK4A136_group*, and *uncultures_bacterium_f_Lachnospiraceae* (Figure 4E).

**Figure 5:**
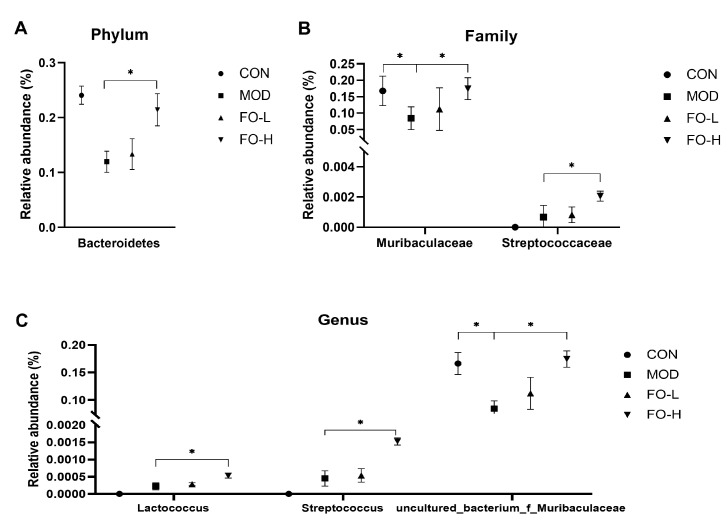
Flaxseed oil altered specific gut microbiota at phylum, family, and genus levels in streptozotocin-induced mice. (**A**–**C**) The relative abundance of gut microbiota at phylum, family, genus levels among groups, respectively. CON, the control group; MOD, the diabetic group; FO-L, 7.0% *w*/*w* flaxseed-oil-added group; FO-H, 10.5% *w*/*w* flaxseed-oil-added group. *, *p* < 0.05.

Furthermore, we compared specific bacteria in different groups at the phylum, family, and genus levels, which indicated that flaxseed oil feeding (10.0% *w*/*w* added in the diet) significantly increased the relative abundance of Bacteroidetes, Muribaculaceae, Streptococcaceae, *Lactococcus*, *Streptococcus*, and *uncultured_bacterium_f_Muribaculaceae* compared to the MOD group (Figure 5).

## 4. Discussion

The findings in the present study indicated that different doses of FO supplementation have differential mechanisms of biological actions in STZ-induced mice. In the present study, FO supplementation at both doses significantly decreased STZ-induced pancreas damage, liver inflammation, and fecal microbiota distribution. The 10.5% *w*/*w* FO supplementation in STZ-induced mice was superior in regulating glucose metabolism in the aspect of the potential of improving the histology of the pancreas and lowering glucose levels and liver glycogen with significance. The 7.0% *w*/*w* FO supplementation in STZ-induced mice showed better anti-inflammatory benefits by decreasing liver TNF-α, IL-1β, and LPS levels. We further found that either 7.0% *w*/*w* or 10.5% *w*/*w* FO feeding significantly modulated liver TLR4 pathways, but 7.0% *w*/*w* FO feeding showed a better effect. Furthermore, it seems that 10.5% *w*/*w* FO feeding showed stronger effects on regulating the relative abundance of fecal microbiota.

Indeed, flaxseed supplementation seemed to improve glycemic control variables and insulin resistance in prediabetes and type 2 diabetes in a systematic review; yet, there is a lack of dose–effect relationship studies [36]. Flaxseed intake was inversely related to total mortality in two prospective cohort studies of the Nurses’ Health Study and the Health Professional Follow-up Study [37]. A previous intake of 15 g of ground raw golden flaxseed (4.8 g of lipids) before breakfast in nineteen men with type 2 diabetes reduced the peak glucose rise in the 2 h AUC glycemic responses by 17% and 24%, respectively [38]. A 2 g intake of FO for the 14-week period tended to decrease fasting blood glucose, C reactive protein, and TNF-α levels in overweight adults with pre-diabetes [39]. The clinical studies showed that FO, flaxseed oil lignan, or flaxseed supplementation markedly reduced serum TNF-α, IL-6, and IL-1β concentrations [40]. So far, most studies involved in FO or flaxseed feeding were conducted in high-fat-diet and STZ-induced type 2 diabetic rodents. In our previous study, we proved that 16.7% w/w-added milled flaxseed ameliorated hepatic inflammation in STZ-induce mice [41], which was observed in the present study as well. An amount of 15% *w*/*w* orally in the feed of flaxseed for 33 days significantly reduced the renal inflammation (IL-1β, IL-6, and NF-κB) in STZ-induced diabetic rats with or without chronic kidney disease [42]. An amount of 10% *w*/*w* FO feeding in SD rats with type 2 diabetes significantly decreased plasma inflammatory indicators (IL-1β, IL-10, IL-17A, IL-6, and TNF-α) and LPS levels, which was similar to the present study where we observed the anti-inflammatory action with both 7% *w*/*w* and 10.5% *w*/*w* FO feeding by reducing hepatic TNF-α and IL-1β levels. An amount of 500 μL of FO feeding significantly decreased hepatic inflammation in obese mice by altering the protein expression of TNF-α, IL-1β, and IL-10 [43]. A 10% FO diet for 35 days ameliorated renal inflammation via reducing the gene expression of IL-6 and NF-κB in STZ-induced diabetic rats [44]. In addition, 10% *w*/*w* FO feeding downregulated the hepatic inflammation gene expression of TNF-α, IL-6, MCP-1, and NF-κB in STZ and nicotinamide-induced diabetic rats [45]. Amounts of 10%, 20%, and 30% *w*/*w* FO replacement for 16 weeks dose-dependently ameliorated systemic insulin resistance and reversed hepatic inflammation via downregulating the gene expression of TNF-α, IL-6, IL-1β, and MCP-1 in high-fat-diet mice [46], which differs from the present study where a lower dose with 7.0% *w*/*w* FO feeding showed a better anti-inflammation potential. Type 1 diabetes shows the innate inflammation involved in a complex interplay of genetic and environmental factors [47]. Previous studies have found that systemic inflammation was less related to complications in type 1 diabetes than in type 2 diabetes [48]. We supposed that in our study, a lower dosage of FO feeding displayed better anti-inflammatory action due to the difference between type 2 and type 1 diabetic mice in terms of the characteristic of inflammation dysfunction. Furthermore, 1.2 FO mL/kg body weight feeding orally reduced oxidative stress in STZ-induced diabetic rats [49]. An amount of 0.714 g/kg body weight/day flaxseed feeding for 12 weeks protected against glucotoxicity by modulating glucose-6-phosphate dehydrogenase and glutathione-s-transferase in the brain of STZ-induced mice [50]. We observed that 10.5% *w*/*w* FO feeding significantly reduced FBG levels within 4 weeks and regulated glucose tolerance, which may be related to its regulation of the abovementioned oxidative stress and glucotoxicity. The progression of type 1 diabetes is always accompanied by inflammation [47]. The liver is the most crucial detoxification and immune organ. LPS, found in the outer membrane of Gram-negative bacteria, plays a crucial role in liver inflammation [51]. In this study, we further explored the underlying mechanisms of FO feeding in terms of anti-inflammation. FO consumption significantly attenuated hepatic LPS levels in STZ-induced mice. TLR4, as the core mediator of inflammation, activates in response to LPS challenge and is expressed in the liver [52]. Accumulating evidence shows that LPS/TLR4 signaling is a key player in liver-related disease [53]. MyD88 as the downstream gene of TLR4 signaling induces p65 phosphorylation, leading to the secretion of inflammatory factors [54]. In the present study, both 7.0% *w*/*w* and 10.5% *w*/*w* FO feeding reversed STZ-induced hepatic inflammation by downregulating LPS/TLR4/MyD88 pathways, which indicated that FO supplementation may act as an anti-inflammatory food in the treatment of type 1 diabetes.

In addition, FO supplementation displayed gut microbiota regulation potential. For example, flaxseed supplementation decreased gut microbiota richness with Firmicutes, Proteobacteria, and Bacteroidetes in the human gut microbiota batch cultures [54]. FO feeding ameliorated high-fat-diet-induced atherosclerosis by reversing the increased ratio of Firmicutes/Bacteroidetes due to gut dysbiosis [35]. A previous study reported that 10% *w*/*w* FO feeding increased the short-chain fatty acids and regulated fecal microbiota by upregulating the relative abundance of Firmicutes and downregulating the relative abundance of Bacteroidetes in fecal intestinal metabolites in type 2 diabetic mice [55]. In the present study, 10.5% *w*/*w* FO feeding significantly increased the relative abundance of Bacteroidetes at the phylum level, which is similar to a previous study. Furthermore, the relative abundance of Muribaculaceae and Streptococcaceae was upregulated at the family and genus levels with FO feeding in STZ-induced mice. The family Muribaculaceae was dramatically more abundant in acarbose-treated mice, which is used as an antidiabetic drug and has displayed the function of longevity [56]. However, the modulation of family and genus Streptococcaceae differs from other studies, which was found to be risen in patients with type 1 diabetes due to different species and the small sample size in the human study [57]. The evidence of effects of FO feeding on gut microbiota in type 1 diabetes is still unclear. Our study provided new ideas on the effects of FO feeding on anti-diabetes. The oral delivery of Lactococcus can prevent type 1 diabetes via ameliorating inflammation [58]. In the present study, 10.5% *w*/*w* FO feeding exhibited the modulation of genus Lactoccus significantly, suggesting its anti-inflammation potential in type 1 diabetes. However, we did not observe dose-dependent effects of FO feeding in type 1 diabetic mice. Together, 10% *w*/*w* FO feeding showed a better microbiota modulation in STZ-induced type 1 diabetic mice.

## 5. Conclusions

In conclusion, FO supplementation displayed the anti-inflammation potential of the liver via LPS/TLR4/MyD88 pathways and the modulation of gut microbiota in STZ-induced mice. Furthermore, different doses of FO supplementation may play different roles.

## Data Availability

The data used to support the findings of this study can be made available by the corresponding author upon request.

## Supplementary Materials

The following supporting information can be downloaded at: https://www.mdpi.com/article/10.3390/foods12173229/s1, Table S1: The ingredients of flaxseed oil fortified diet in different groups (g/100 g).
