# Supplementary material for: Beneficial Effects of Dietary Flaxseed Oil through Inflammation Pathways and Gut Microbiota in Streptozotocin-Induced Diabetic Mice
Source: Foods. 2023 Aug 28;12(17):3229. doi: 10.3390/foods12173229 (PMC10487211; doi:10.3390/foods12173229)
Supplement: Supplementary file 1 [file foods-12-03229-s001.zip › foods-2560192-supplementary.pdf]

foods

Table S1. The ingredients of flaxseed oil fortified diet in different groups (g/100g).

Ingredient	Group			
	CON	MOD	FO-H	FO-L
Flaxseed oil			10.5	7.0
Casein	20.00	20.00	20.00	20.00
Corn starch	39.75	39.75	36.25	39.74
Dextrin	13.20	13.20	13.20	13.20
Sucrose	10.00	10.00	10.00	10.00
Fiber	5.00	5.00	5.00	5.00
Soybean oil	7.00	7.00	-	
Complex minerals	3.50	3.50	3.50	3.50
L-cystine	0.30	0.30	0.30	0.30
Complex vitamins	1.00	1.00	1.00	1.00
Choline bitartrate	0.25	0.25	0.25	0.25
Tert-butyl hydroquinone	0.0014	0.0014	0.0014	0.0014
Energy (kcal/100g)	395			

CON, the control group; MOD, the diabetic group; FO, flaxseed oil (15 mL, equal as 14g, providing 120 Calories and including 1.5g of n-6 polyunsaturated fatty acid, 7g of α-linolenic acid, 2.5 g of monounsaturated fatty acid and 1.5g of saturated fatty acid)
